# An Exploratory Study: Can Vision-Enabled Large Language Models Interpret Selected Static Urologic Images?

**DOI:** 10.7759/cureus.117477

**Published:** 2026-10-05

**Authors:** Max S Yudovich, Sasha A Mainer, Taylor L Lan, Jay D Raman

**Affiliations:** 1 Urology, Penn State Health Milton S. Hershey Medical Center, Hershey, USA; 2 Urology, Mercer University School of Medicine, Columbus, USA

**Keywords:** artificial intelligence, large language models, medical imaging, medical interpretation, urology

## Abstract

Introduction

Vision-enabled large language models (LLMs) are increasingly capable of interpreting medical images. However, performance across common urologic imaging modalities remains poorly defined. We compared four publicly available vision-enabled LLMs on their ability to identify imaging modalities and interpret selected static urologic images at a single point in time.

Methods

This exploratory observational study evaluated 16 selected static urologic images comprising radiography, US, computed tomography (CT), and MRI (four images per modality). Four publicly available vision-enabled LLMs (ChatGPT-4o (OpenAI, Inc., San Francisco, CA, USA), Grok 2.0 beta (SpaceXAI LLC, San Francisco, CA, USA), Gemini 1.5 (Google DeepMind, London, UK), and Copilot Azure AI Vision 3.2 (Microsoft Corporation, Redmond, WA, USA)) identified the imaging modality and interpreted each image before and after the addition of an arrow highlighting the principal abnormality. Each model was accessed through its public consumer interface between May 1 and May 10, 2025, and generated a single response per image per condition. Responses were graded by one unblinded investigator using a predefined 5-point Likert scale based on interpretation accuracy.

Results

ChatGPT and Grok correctly identified the imaging modality in all 16 images (100%). Copilot correctly identified 10 of 16 images (62.5%), misclassified two (12.5%), and declined to interpret four (25.0%; three US and one MRI) because of content-filtering restrictions. Gemini declined to interpret all images. Among the 12 images interpreted by all three models, median interpretation scores without arrow context were 4.0 (IQR 3.0-5.0) for ChatGPT, 4.0 (IQR 3.0-4.3) for Grok, and 2.0 (IQR 2.0-4.0) for Copilot (Friedman P = 0.004). Addition of contextual arrows was not associated with a statistically detectable change in interpretation accuracy for any model (all P ≥ 0.20), although the CIs were wide and a clinically meaningful effect cannot be excluded.

Conclusions

Performance varied substantially among publicly available vision-enabled LLMs. ChatGPT and Grok achieved higher interpretation scores than Copilot on this limited exploratory image set, whereas Copilot and Gemini were constrained by content moderation policies. These findings describe model behavior on selected static images at a single point in time and do not establish diagnostic accuracy or readiness for clinical use. Larger studies using standardized datasets, repeated model runs, and independent blinded expert evaluation are needed to define the role of vision-enabled LLMs in urologic image interpretation.

## Introduction

AI has rapidly permeated clinical medicine, with usage among physicians having increased by 78% since 2023, propelled by iterative advances in machine- and deep-learning architectures that ingest vast, heterogeneous datasets and solve increasingly complex problems [1,2]. Imaging is a natural proving ground for these systems. High-resolution digital data are abundant, and accurate interpretation directly influences patient outcomes. Specifically, AI algorithms have achieved promising performance in breast cancer screening [3], magnetic resonance detection of meniscal injury [4], and pediatric abdominal imaging [5].

Urologic practice, where surgical decision-making often hinges on nuanced radiologic detail, has embraced similar tools. Early applications include ambient “AI scribes” in outpatient clinics [6], automated Gleason grade grouping to refine prostate cancer risk stratification [7], multimodal platforms that integrate clinical, pathologic, and radiologic variables to guide oncologic therapy [8], and computer-vision enhancements that improve lesion detection during cystoscopy and transurethral resection [9]. Yet, broad surgical adoption remains cautious. Concerns persist regarding dataset bias and external validity. Importantly, users question the reliability of AI when tasked with independent image interpretation, particularly across diverse modalities encountered in urologic oncology and stone surgery.

To clarify current capabilities, we evaluated four publicly available large language models (LLMs) with vision functionality: ChatGPT-4o (OpenAI, Inc., San Francisco, CA, USA), Grok 2.0 beta (SpaceXAI LLC, San Francisco, CA, USA), Gemini 1.5 (Google DeepMind, London, UK), and Copilot Azure AI Vision 3.2 (Microsoft Corporation, Redmond, WA, USA), using a curated series of plain-film (KUB), US, CT, and MRI urologic studies. Each model was scored for correct modality recognition and identification of critical findings, both with and without a contextual visual cue. The primary objective was to compare interpretation performance across models on this image set. Secondary objectives were to describe imaging modality recognition, response refusal rates, differences across imaging modalities, and the effect of arrow annotation. This is a time-specific feasibility comparison on a small, curated image set; it was not designed to establish diagnostic accuracy or readiness for clinical use, and the results should not be read as a clinical benchmark.

The abstract of this manuscript was selected, and a poster was presented at the American College of Surgeons Clinical Congress on October 5, 2025.

## Materials and methods

This exploratory observational study evaluated the ability of publicly available vision-enabled LLMs to identify imaging modalities and interpret common urologic imaging studies. Four commercially available LLMs with image interpretation capabilities were evaluated: ChatGPT-4o, Grok 2.0 beta, Gemini 1.5, and Copilot Azure AI Vision 3.2. Each model was accessed through its publicly available consumer-facing web interface between May 1 and May 10, 2025, using standard (non-enterprise) accounts; no application programming interface was used, and no system instruction or configurable generation parameter (for example, temperature or reasoning mode) was set beyond each platform default. These systems were selected because they were publicly accessible platforms that accepted image input at the time of testing, and they should not be assumed to be architecturally equivalent vision-enabled LLMs. Differences in underlying architecture, image-processing pipeline, user interface, and content-moderation policy may all contribute to the performance differences reported here. Historical build identifiers, subscription-tier settings, and regional routing were not prospectively recorded and cannot be reliably reconstructed.

A test set of 16 Creative Commons Attribution-ShareAlike 3.0 images was assembled by convenience sampling of publicly available sources to span four imaging modalities commonly encountered in clinical urologic practice. The image set included four radiographs (one IV pyelogram and three plain abdominal radiographs), four US images, four CT images, and four MRI studies (Table 1). Images were selected by the investigators to provide a heterogeneous exploratory sample of benign and malignant urologic findings; four images per modality cannot represent the spectrum, difficulty, or technical variability of imaging encountered in practice, and the set was not assembled to be statistically representative. Each image was a single static frame rather than a complete DICOM series, and no clinical history, laboratory data, or prior imaging accompanied any image. One image, a normal urinary bladder US, was included as a normal study; a systematically assembled cohort of normal or negative control images was not included, and specificity, false-positive rates, and the propensity for overcalling therefore could not be estimated. For each image, a single target finding, which could be either a pathologic finding or normal anatomy, was defined in advance and served as the reference standard for scoring (Table 1).

**Table 1:** The 16 selected static urologic images used in this study, four from each of four imaging modalities, with the predefined target finding that served as the reference standard for scoring.

Imaging modality	Predefined target finding
Radiography	IV pyelogram
	Kidney stone
	Ureteral stent
	Bladder stones
US	Kidney stone
	Hydrocele
	Normal urinary bladder (no abnormality)
	Hydronephrosis
CT	Emphysematous pyelonephritis
	Renal mass
	Urolithiasis
	Bladder tumor
MRI	Renal mass
	Bladder tumor
	Retroperitoneal hematoma
	PI-RADS 5 of the left lobe

Each unannotated image was submitted individually to each LLM in a new session with the standardized prompt, “Identify the imaging modality and describe the findings in the image.” Imaging modality identification (radiography, US, CT, or MRI) was recorded separately as a categorical outcome (correct, incorrect, or refused). The image was then annotated with a single red arrow placed adjacent to the predefined target finding, with the arrowhead directed toward the region of interest and positioned so that it did not overlie the target finding or obscure relevant anatomy, and was resubmitted with the prompt, “Identify the imaging finding indicated by the red arrow” (Figure 1). The second prompt was intentionally more targeted than the first and was not an identical repetition of it. Source images were retained at their native resolution, which varied across the 16 images; the exact arrow dimensions and the annotation software used were not prospectively recorded. For every image, the unannotated condition was evaluated before the annotated condition, and presentation order was neither randomized nor counterbalanced. Each model generated one response per image under each condition; repeated independent runs were not performed, so within-model response variability could not be assessed. No investigator-authored system instructions were used beyond the default behavior of each platform.

**Figure 1:**
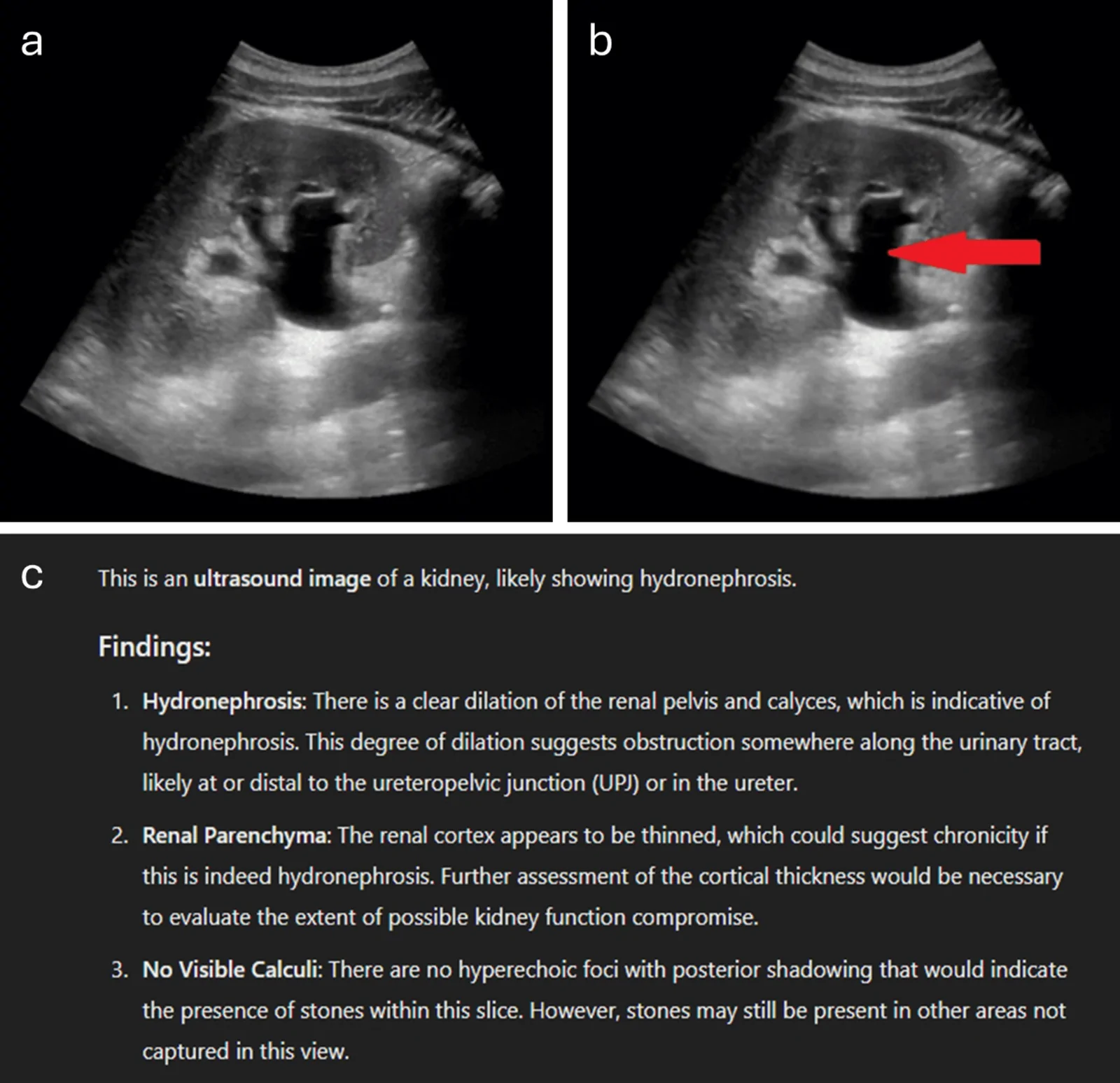
ChatGPT US interpretation. Example of a US image of hydronephrosis without (a) and with (b) arrow context, including an AI interpretation from ChatGPT (c).

Interpretation quality was evaluated independently from imaging modality identification using a predefined five-point Likert scale (Table 2), where a score of 1 represented a completely incorrect interpretation and a score of 5 represented accurate identification and interpretation of the principal imaging finding. Imaging modality identification was not incorporated into the Likert score and was analyzed separately. The rubric was developed by the investigators for this study and has not been externally validated; it was adapted in concept from published ordinal scales used to rate radiology report quality (14) and was intended to capture, in a single ordinal score, both descriptive completeness and identification of the predefined target finding. Because the scale collapses these dimensions into one value, it does not separately quantify diagnostic correctness, descriptive completeness, and clinically consequential error, and it does not explicitly penalize unsupported additional findings or hallucinated content; a response that identified the target finding while also reporting unsupported findings could therefore still receive a high score. All responses were graded by a single investigator (MSY) using the predefined rubric. The grader was not blinded to model identity, arrow condition, or the predefined target finding. Because a second independent reviewer did not score the responses, inter-rater agreement could not be assessed, and no adjudication process was required. Single unblinded grading is a substantive source of subjective bias that uniform application of predefined criteria mitigates but does not eliminate.

**Table 2:** Likert scale value and corresponding criteria descriptions for grading interpretation of images with and without context.

Likert scale value	Descriptions
1	Completely incorrect description
2	Partially describes the image and misses critical findings
3	Thoroughly describes the image but misses critical findings
4	Completely describes the image with partially incorrect or not definitive critical findings
5	Completely describes the image, including the critical finding

Given the exploratory design, limited sample size, and ordinal nature of the five-point Likert scale, results are summarized primarily using descriptive statistics, including medians and IQRs. Imaging modality identification is reported as frequencies and percentages with 95% Wilson CIs. Within-model comparisons of interpretation scores before and after the addition of arrow context were performed using exact Wilcoxon signed-rank tests, with the matched-pairs rank-biserial correlation (r) reported as an effect size and a bootstrap 95% CI for the median paired difference. Overall differences in interpretation scores among LLMs were evaluated using Friedman tests with Kendall’s W as an effect size, followed by post hoc pairwise exact Wilcoxon signed-rank tests. Because three pairwise comparisons were performed within each arrow condition, post hoc P values were adjusted using the Holm-Bonferroni method; both unadjusted and adjusted values are reported. Comparisons across imaging modalities were performed using Kruskal-Wallis tests with epsilon-squared (H divided by n - 1) as an effect size. Between-model comparisons were restricted to the 12 images for which ChatGPT, Grok, and Copilot each produced an interpretable response, and a sensitivity analysis retained all 16 images and assigned refused responses the lowest score of 1, treating refusal as failure to produce an interpretation. All analyses are exploratory. Given the very small sample and the number of comparisons performed, interpretation emphasizes effect estimates and their uncertainty rather than statistical significance, and exact two-sided P values are reported as descriptive quantities rather than as tests of prespecified hypotheses. Analyses were performed using IBM SPSS Statistics for Windows, version 32.0 (released 2026; IBM Corp., Armonk, NY, USA).

All images were publicly available, contained no patient identifiers, and were obtained from Creative Commons Attribution-ShareAlike 3.0 sources. Because the study involved no human participants and no identifiable private information, the institution’s human research protection program determined that it did not constitute human subjects research, and an institutional review board review was not sought. No image preprocessing was performed other than the addition of the red arrow annotation described above.

## Results

ChatGPT and Grok correctly identified the imaging modality for all 16 images (100%). Copilot correctly identified the modality in 10 images (62.5%), misclassified two (12.5%), and declined to interpret four images in total (25.0%): three US studies (kidney stone, bladder, and hydronephrosis) and one MRI study (renal mass). Both misclassified images were MRI studies (bladder tumor and retroperitoneal mass). Modality identification accuracy was 100% (95% CI 80.6-100%) for ChatGPT and Grok and 62.5% (95% CI 38.6-81.5%) for Copilot. Gemini declined to interpret all 16 submitted images (refusal rate 100%) and was therefore excluded from subsequent interpretation analyses. All refusals were returned as content-policy messages rather than as failed or empty interpretations and therefore reflect platform moderation behavior rather than a demonstrated inability to analyze the image; the underlying image-analysis capability of Copilot and Gemini for the refused images could not be assessed. Image-level modality classifications, interpretation scores, and refusals for every image and model are reported in Table 3.

**Table 3:** Image-level results for all 16 images. For each model, the entry gives imaging modality identification (C = correct, I = incorrect, R = refused) followed by the interpretation score without arrow context/with arrow context. R denotes a content-policy refusal, for which no interpretation score could be assigned. Gemini refused all 16 images under both conditions.

Image	Modality	Predefined target finding	ChatGPT	Grok	Copilot	Gemini
1	Radiography	IV pyelogram	C; 5/4	C; 5/3	C; 5/4	R
2	Radiography	Kidney stone	C; 4/5	C; 4/2	C; 4/5	R
3	Radiography	Ureteral stent	C; 5/5	C; 4/5	C; 4/5	R
4	Radiography	Bladder stones	C; 5/5	C; 3/4	C; 4/3	R
5	US	Kidney stone	C; 3/5	C; 2/2	R	R
6	US	Hydrocele	C; 2/2	C; 3/2	C; 2/1	R
7	US	Normal urinary bladder	C; 5/5	C; 4/3	R	R
8	US	Hydronephrosis	C; 5/5	C; 4/4	R	R
9	CT	Emphysematous Pyelonephritis	C; 3/5	C; 3/4	C; 2/3	R
10	CT	Renal mass	C; 4/3	C; 5/5	C; 2/5	R
11	CT	Urolithiasis	C; 3/3	C; 3/4	C; 2/4	R
12	CT	Bladder tumor	C; 3/5	C; 2/3	C; 2/4	R
13	MRI	Renal mass	C; 3/5	C; 5/4	R	R
14	MRI	Bladder tumor	C; 5/5	C; 4/2	I; 1/1	R
15	MRI	Retroperitoneal hematoma	C; 4/2	C; 4/5	I; 1/1	R
16	MRI	PI-RADS 5 of the left lobe	C; 5/5	C; 5/3	C; 2/2	R

Interpretation accuracy varied among the LLMs. Without arrow context, the median Likert interpretation scores were 4.0 (IQR 3.0-5.0) for ChatGPT, 4.0 (IQR 3.0-4.3) for Grok, and 2.0 (IQR 2.0-4.0) for Copilot (n = 12 interpretable responses). Following the addition of arrow context, median scores were 5.0 (IQR 3.8-5.0) for ChatGPT, 3.5 (IQR 2.8-4.0) for Grok, and 3.5 (IQR 1.8-4.3) for Copilot (Figures 2, 3).

**Figure 2:**
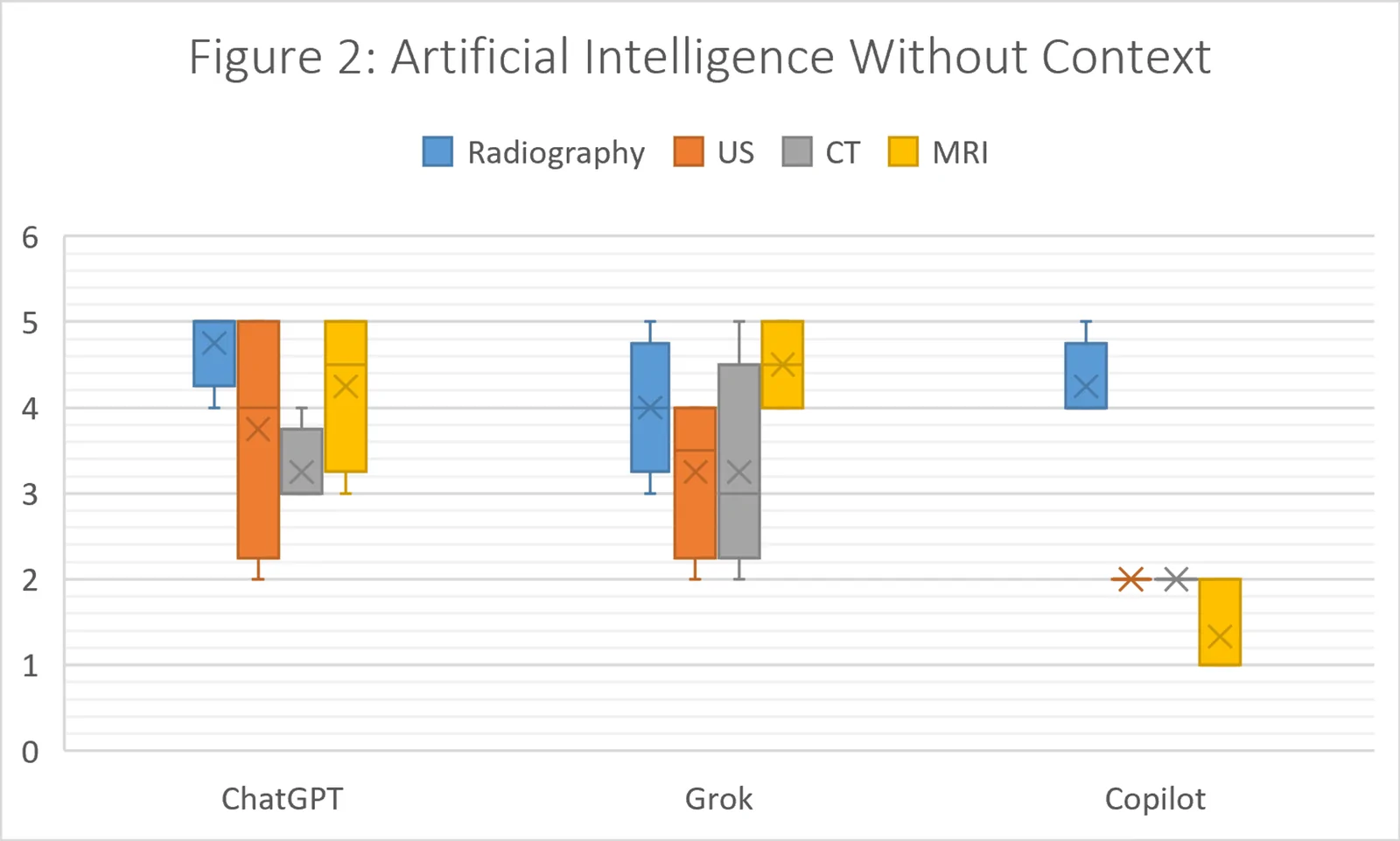
Box plot using individual image-level Likert scores for each AI tool without red arrow context, where n = 12 for Copilot. Gemini was excluded due to refusal.

**Figure 3:**
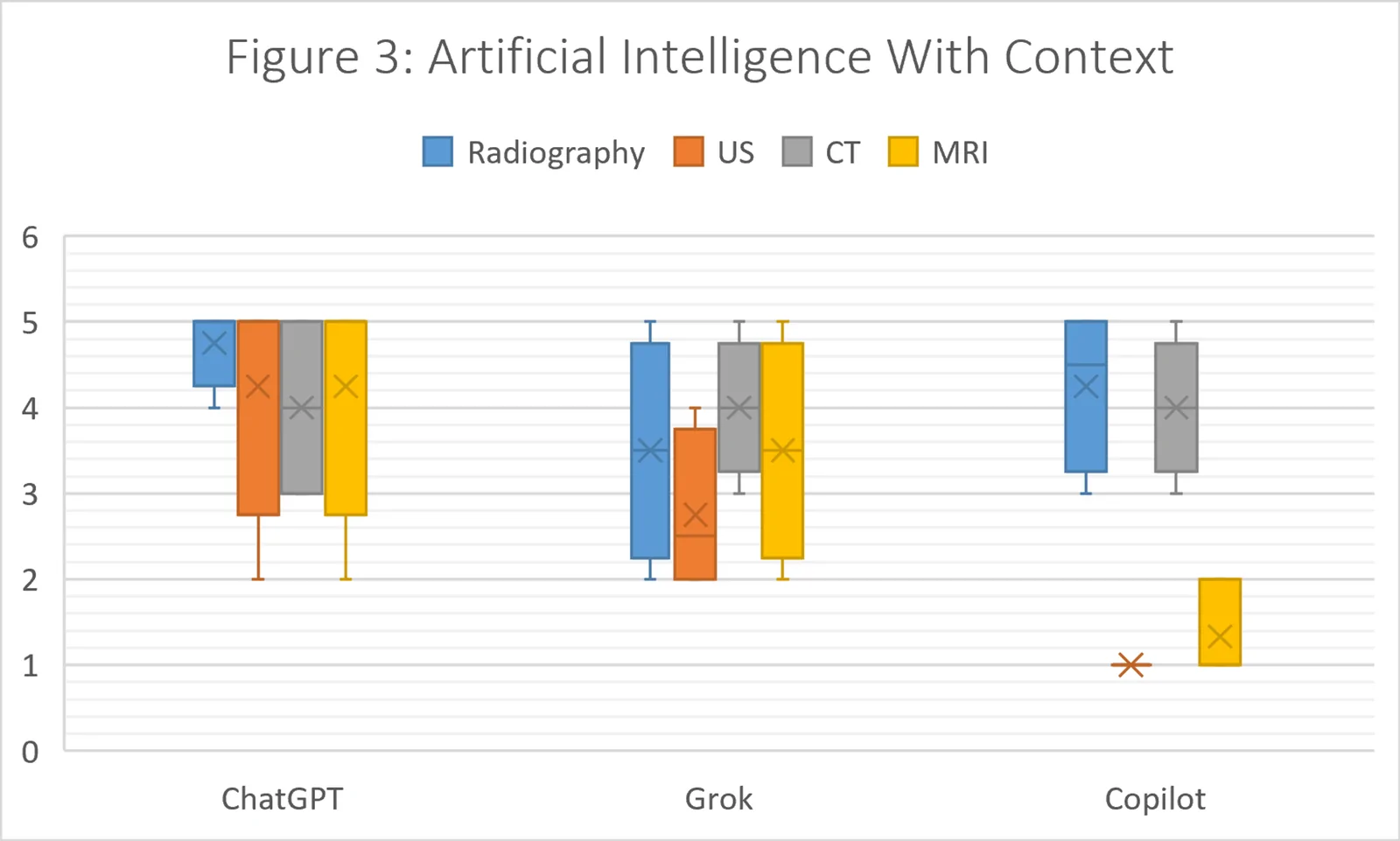
Box plot using individual image-level Likert scores for each AI tool with the addition of red arrow context, where n = 12 for Copilot. Gemini was excluded due to interpretation refusal.

No statistically detectable change in overall interpretation accuracy followed the addition of arrow context for ChatGPT (median paired difference 0, bootstrap 95% CI 0 to +1.0; rank-biserial r = +0.44; exact P = 0.31), Grok (median paired difference 0, 95% CI -1.0 to +1.0; r = -0.34; P = 0.31), or Copilot (median paired difference +0.5, 95% CI -0.5 to +1.5; r = +0.53; P = 0.20). Scores improved for 5, 6, and 6 images and worsened for 3, 7, and 3 images for ChatGPT, Grok, and Copilot, respectively. These intervals are wide and encompass differences that would be clinically meaningful; these data therefore show an absence of detectable benefit rather than evidence that arrow annotation confers no benefit. Results were similar when analyses were stratified by imaging modality (all exact P ≥ 0.13), although each stratum contained at most four images and these comparisons are severely underpowered.

To permit paired comparisons, analyses between LLMs were restricted to the 12 images for which ChatGPT, Grok, and Copilot each generated an interpretable response (four radiography, one US, four CT, and three MRI). The four excluded images were those Copilot refused, three of which were US studies; this complete-case restriction removes the images on which one model was unable to respond and may therefore understate between-model differences. Without arrow context, interpretation scores differed among the three models (Friedman χ² = 10.97, df = 2, P = 0.004, n = 12; Kendall’s W = 0.46). In post hoc pairwise comparisons, ChatGPT scored higher than Copilot (median difference +1.25, r = +1.00; exact P = 0.004, Holm-adjusted P = 0.012), and Grok scored higher than Copilot (median difference +1.00, r = +0.86; P = 0.039, Holm-adjusted P = 0.078), whereas ChatGPT and Grok did not differ detectably (median difference 0, r = +0.43; P = 0.44, Holm-adjusted P = 0.44).

Following the addition of arrow context, interpretation scores did not differ detectably among the three models (Friedman χ² = 3.74, df = 2, P = 0.15, n = 12; Kendall’s W = 0.16). Pairwise comparisons likewise showed no detectable differences between ChatGPT and Grok (median difference +0.50, r = +0.36; P = 0.38, Holm-adjusted P = 0.75), ChatGPT and Copilot (median difference +1.00, r = +0.62; P = 0.13, Holm-adjusted P = 0.39), or Grok and Copilot (median difference +0.50, r = +0.29; P = 0.50, Holm-adjusted P = 0.75).

In the sensitivity analysis that retained all 16 images and scored refusals as 1, the median interpretation score for Copilot was 2.0 (IQR 1.0-2.5) without arrow context and 2.5 (IQR 1.0-4.0) with arrow context. Differences among models were larger than in the complete-case analysis both without arrow context (Friedman χ² = 17.43, df = 2, P < 0.001; ChatGPT versus Copilot P < 0.001, Bonferroni-adjusted P < 0.001; Grok versus Copilot P = 0.002, adjusted P = 0.007) and with arrow context (Friedman χ² = 9.93, df = 2, P = 0.007; ChatGPT versus Copilot P = 0.008, adjusted P = 0.024). Excluding refusals therefore understates the practical performance gap between platforms, and the refusal rate is reported here as an availability outcome in its own right.

Differences in interpretation accuracy across imaging modalities were not statistically detectable without arrow context for ChatGPT (Kruskal-Wallis H = 4.88, df = 3, P = 0.18, n = 16; ε² = 0.33) or Grok (H = 4.50, df = 3, P = 0.21, n = 16; ε² = 0.30). For Copilot, scores differed across modalities (H = 9.78, df = 3, P = 0.02, n = 12; ε² = 0.89), but this comparison rests on group sizes of four, one, four, and three images and is not interpretable. Mean scores without arrow context were highest for radiography for ChatGPT (4.75) and Copilot (4.25) but highest for MRI for Grok (4.50), and the lowest-scoring modality differed by model. With at most four images per modality, all modality comparisons are severely underpowered, and no modality-specific conclusion is supported.

## Discussion

In this exploratory study, we evaluated the performance of four publicly available vision-enabled LLMs across a selected set of 16 static urologic images. ChatGPT and Grok consistently identified the imaging modality and generally achieved higher interpretation scores than Copilot. Gemini declined to interpret all submitted images. Although the limited sample size precludes broad conclusions regarding diagnostic performance, these findings suggest that the multimodal LLMs available in May 2025 differed substantially in both observed interpretation performance and practical availability. Because each model was queried once per image and these platforms are updated frequently, the results describe a time-specific snapshot rather than a stable property of any platform.

One of the most notable findings was the influence of platform-specific content moderation policies. While ChatGPT and Grok generated responses for every submitted image, Copilot declined to interpret four studies. Gemini declined all image interpretation requests despite recent publications claiming that the model was programmed with medical imaging analysis, dialogue, and feedback capabilities [10]. These refusals occurred despite the use of publicly available, non-identifiable medical images and limited the models' clinical utility independent of their image interpretation capabilities. Consequently, future evaluations of vision-enabled LLMs should consider response refusal rates and content moderation behavior in addition to traditional measures of diagnostic accuracy, as these factors may substantially affect real-world implementation. Because a refusal returns a content-policy message rather than an attempted interpretation, refusal rate measures platform governance rather than image-analysis capability, and the two should be reported and interpreted separately.

The addition of a simple visual cue in the form of an arrow highlighting the principal abnormality did not significantly improve interpretation accuracy for any evaluated model. Another study went further to include attention heatmaps in detecting benign or malignant pathology but received no mechanistic explanation for model labeling [11]. These findings differ from those of Huppertz et al., who reported improved diagnostic performance after providing ChatGPT-4 with accompanying clinical vignettes during interpretation of musculoskeletal radiographs [12]. Unlike a clinical history, which provides contextual information regarding symptoms, disease probability, and clinical presentation, an arrow supplies only spatial guidance. These findings suggest that current multimodal LLMs may derive greater benefit from clinical context than from simple visual annotation alone.

Moreover, one study found an increase in diagnostic accuracy for clinicians using standard MRI LLMs accompanied by a respiratory failure clinical vignette. This was seen with AI predictions and with AI predictions plus explanations [13]. Future investigations should evaluate clinician expertise and combinations of imaging, clinical history, and laboratory data in tandem to better approximate real-world diagnostic workflows.

ChatGPT achieved a median interpretation score of 4.0 without and 5.0 with arrow context on this image set, and Grok scored similarly without arrow context. A published report of radiology resident reporting performance using a different ordinal instrument described a mean score of less than 4 per area prior to dedicated training [14]; that study used different images, a different task, a different scoring instrument, and a different reference standard, and the present study reports medians rather than means. The two values are therefore not comparable, and we make no claim that any model matched or exceeded trainee performance. A direct comparison would require presenting the same images and the same scoring rubric to both models and clinicians. For the same reasons, these data do not support the use of vision-enabled LLMs for triage, second reading, or high-volume clinical support, none of which was evaluated here.

With reports of PGY-4 radiology residents averaging a Likert score of 3.8 after dedicated training sessions, ChatGPT’s median Likert score of ≥ 4, both with and without the arrow, shows promise as it exceeds the threshold many institutions expect from trainee preliminary interpretations [13]. Grok trailed only marginally. This finding reinforces the notion that multimodal LLMs are advancing quickly and could already provide useful triage, comparison, or double-reading support for urologists managing high imaging volumes. Although further studies using the same images for LLMs and resident physicians would be needed to make direct comparisons, these preliminary findings warrant further investigation.

Our findings contribute to the growing literature evaluating LLMs for medical image interpretation. Several previously published evaluations of multimodal LLMs in radiology have examined a single imaging modality or organ system [11,12], whereas the present study spans four modalities routinely encountered in urologic practice, including radiography, US, CT, and MRI. We did not conduct a systematic review of prior multimodal medical imaging studies, and we therefore describe this multimodality scope as a feature of the present design rather than as an established novelty claim. Additionally, we exclusively utilized Creative Commons images to avoid copyright restrictions and enhance reproducibility. Although the image set was intentionally limited and used one selected imaging frame, the inclusion of multiple imaging modalities provides a broader assessment of current multimodal LLM capabilities than studies focused on a single imaging type.

It should be noted that Jabbour et al. reported a significant decrease in clinician accuracy when AI was purposely biased against elderly, obese, and other factors [13]. Other reports show similar bias against commonly underserved demographics, even when these protected attributes were not provided [15]. It was also discovered that LLMs will use noncontributory imaging features in interpretations, a phenomenon termed “shortcut learning” [15]. No model can be assumed to be free of bias, and bias cannot be reliably excluded by model selection alone. Mitigation instead requires external validation in the intended population, prespecified subgroup performance testing, ongoing post-deployment monitoring, and institutional governance covering data handling and patient privacy.

Several limitations warrant consideration. First, this was an exploratory pilot study evaluating only 16 representative images, limiting statistical power and generalizability. Second, responses were graded by a single reviewer using a standardized scoring rubric, introducing the potential for subjective bias despite consistent application of predefined grading criteria. Third, publicly available Creative Commons images may not fully reflect the diversity, complexity, or technical variability encountered in routine clinical practice. Fourth, publicly available LLMs are updated frequently, and performance may differ as models evolve over time. Finally, proprietary content moderation systems are not transparent, making it difficult to determine why certain images were refused despite lacking identifiable patient information.

Several further limitations warrant emphasis. The stimuli were isolated static frames rather than complete DICOM examinations, and no clinical history, laboratory data, or prior imaging was provided, so the task differs materially from clinical image interpretation. The image set contained a single normal study and no systematically assembled negative controls, so specificity, false-positive rates, and overcalling could not be assessed. The reference standard was the target finding specified in advance by the investigators rather than an independently adjudicated diagnosis. Each model was queried only once per image, so response stability across repeated runs is unknown, and individual responses may not be reproducible. Scoring was performed by one investigator who was not blinded to model identity, arrow condition, or the expected finding, and inter-rater agreement was not assessed. The scoring rubric was developed for this study, has not been validated, and does not separately penalize hallucinated or unsupported findings.

The fixed presentation sequence, in which each unannotated image preceded its annotated counterpart, may also have introduced order or carryover effects, so differences between conditions cannot be attributed solely to the additional spatial guidance. Restricting between-model comparisons to the 12 images with complete responses may have introduced selection bias by removing images that one platform declined, which is why the sensitivity analysis treating refusal as failure is reported alongside the complete-case results. The complete-case sample of 12 images, with as few as one image in some modality strata, limits the reliability of every inferential statistic reported here, and the modality-stratified comparisons in particular should be regarded as hypothesis-generating only.

Despite these limitations, this study provides a contemporary comparison of publicly available vision-enabled LLMs across multiple urologic imaging modalities and highlights practical considerations beyond interpretation accuracy alone. As multimodal LLMs continue to evolve, future studies should incorporate larger, standardized image datasets; repeated independent model runs; at least two blinded expert reviewers with reported inter-rater agreement; a prespecified adjudication process; clinically representative imaging including normal and negative controls; and integration of clinical information to better define their potential role in urologic practice.

## Conclusions

Vision-enabled LLMs varied widely in their responses to a selected set of static urologic images. ChatGPT and Grok produced interpretable responses for every image and achieved higher interpretation scores than Copilot in this limited exploratory dataset, whereas Copilot and Gemini were constrained by content moderation policies that limited their practical availability. These findings characterize model behavior on selected individual images at a single point in time; they do not establish diagnostic accuracy, clinical safety, or readiness for clinical decision support. Although these findings support continued investigation of multimodal LLMs for medical image interpretation, larger validation studies using standardized datasets and independent expert evaluation are required before these systems can be considered for routine clinical decision support.

## Appendices

https://docs.google.com/document/d/10FdWu3-dXDCIKN3vv3wWS7Ja64JOQnGOnGCvOqGOTE8
